# Supplementary material for: Effect of Different Direct Compaction Grades of Mannitol on the Storage Stability of Tablet Properties Investigated Using a Kohonen Self-Organizing Map and Elastic Net Regression Model
Source: Pharmaceutics. 2020 Sep 18;12(9):886. doi: 10.3390/pharmaceutics12090886 (PMC7559487; doi:10.3390/pharmaceutics12090886)
Supplement: Supplementary file 1 [file pharmaceutics-12-00886-s001.zip › pharmaceutics-922795-supplementary.pptx]

## Slide 1
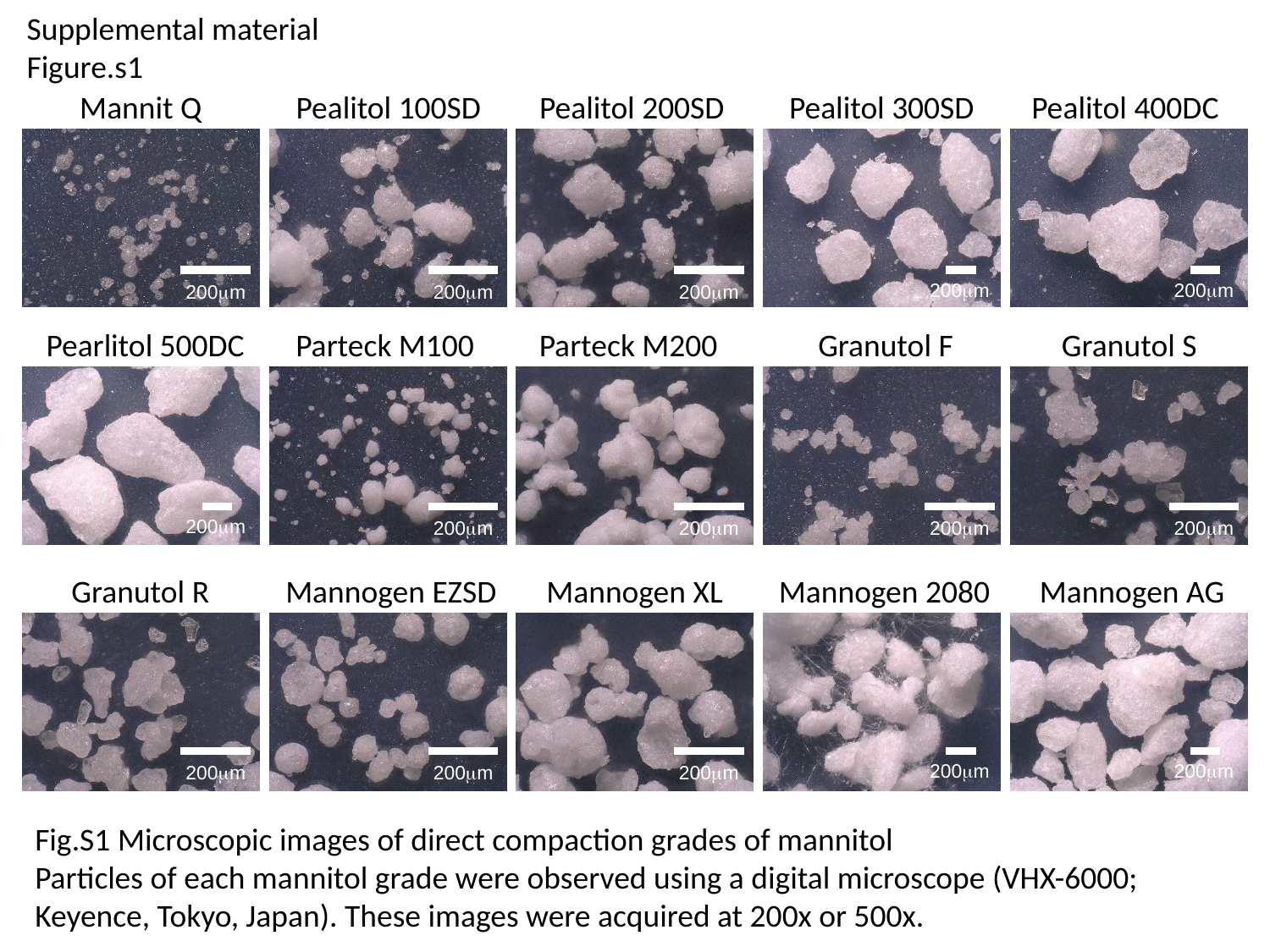

Supplemental material
Figure.s1
Mannit Q
Pealitol 100SD
Pealitol 200SD
Pealitol 300SD
Pealitol 400DC
200mm
200mm
200mm
200mm
200mm
Pearlitol 500DC
Parteck M100
Parteck M200
Granutol F
Granutol S
200mm
200mm
200mm
200mm
200mm
Granutol R
Mannogen EZSD
Mannogen XL
Mannogen 2080
Mannogen AG
200mm
200mm
200mm
200mm
200mm
Fig.S1 Microscopic images of direct compaction grades of mannitol
Particles of each mannitol grade were observed using a digital microscope (VHX-6000; Keyence, Tokyo, Japan). These images were acquired at 200x or 500x.

## Slide 2
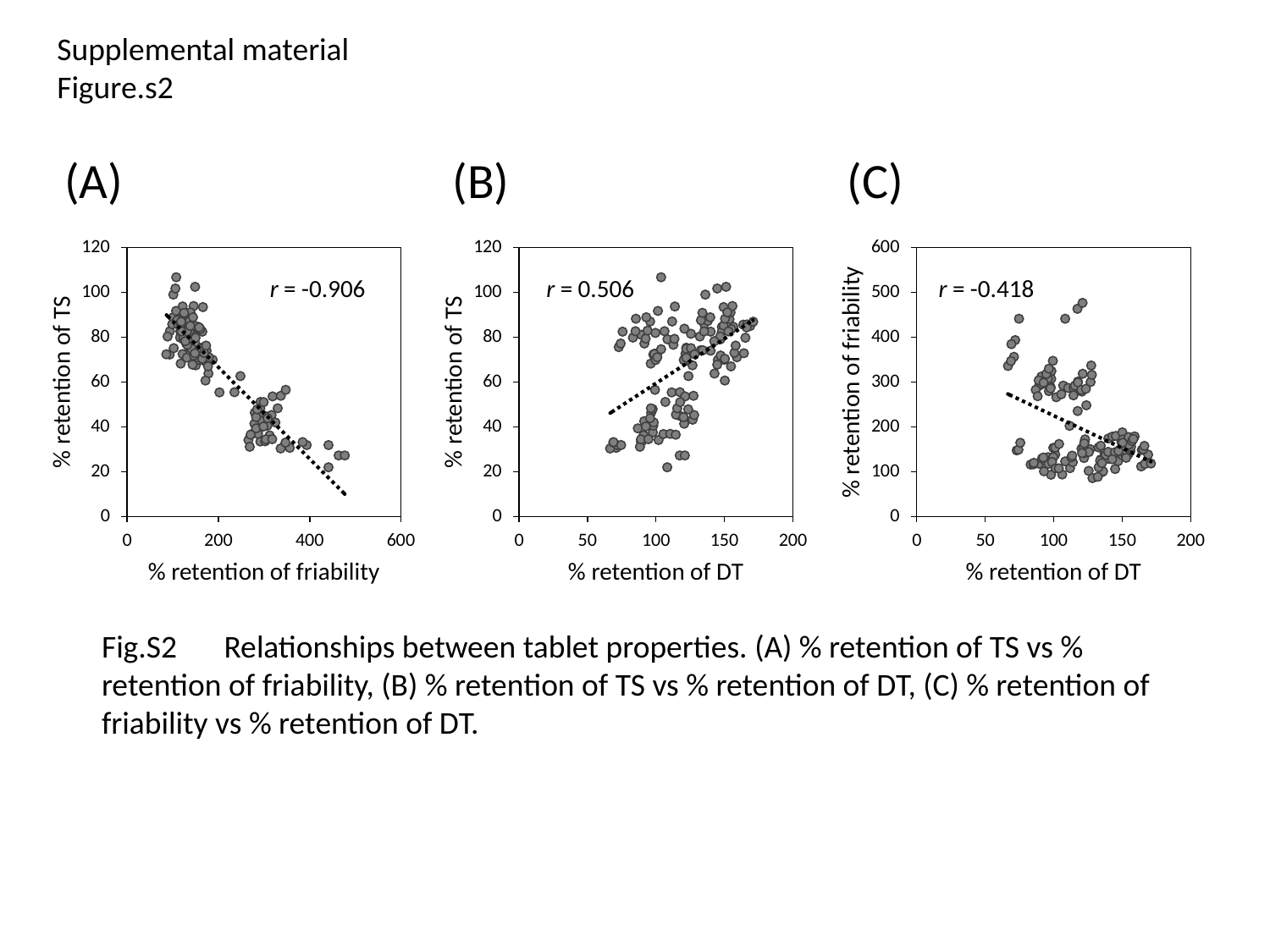

Supplemental material
Figure.s2
(A)
(B)
(C)
r = -0.906
r = 0.506
r = -0.418
Fig.S2　Relationships between tablet properties. (A) % retention of TS vs % retention of friability, (B) % retention of TS vs % retention of DT, (C) % retention of friability vs % retention of DT.
